# 667. Vascularized Composite Allotransplantation a Decade Later: A Contemporary Analysis of National Outcomes

**DOI:** 10.1093/jbcr/irag033.533

**Published:** 2026-04-06

**Authors:** Sophia A Zabul, Sovan Ven, Kevin Vargas, Anthony Shadid, Abbas Rana

**Affiliations:** Baylor College of Medicine, Houston, TX; Baylor College of Medicine, Houston, TX; Baylor College of Medicine, Houston, TX; Baylor College of Medicine, Houston, TX; Baylor College of Medicine, Houston, TX

## Abstract

**Introduction:**

This study provides the most contemporary analysis of the United States vascularized composite allograft (VCA) experience, utilizing the national Organ Procurement and Transplantation Network (OPTN) database for all transplants performed through 2023.

**Methods:**

From the Organ Procurement and Transplantation Network (OPTN)/United Network for Organ Sharing (UNOS) Standard Transplant Analysis and Research (STAR) registry, we identified 476 VCA transplant records and analyzed graft survival using Kaplan–Meier methods.

**Results:**

The field has evolved, with upper limb (46.0%), uterus (22.3%), and face (21.2%) transplants being the most common procedures. Overall 5-year graft survival was 96.1%. Graft survival was paradoxically worse in the modern era (2016-Present, *p*=.0003) and differed significantly by VCA type (*p*<.0001). Notably, upper limb allografts demonstrated 100% graft survival. Multivariable modeling, performed on non-upper limb VCAs, revealed that uterus transplants had a significantly higher risk of graft failure compared to face transplants (HR 7.33, *p*=.008). Additionally, increasing donor age showed a trend towards being a risk factor for graft failure (HR 1.05, *p*=.066).

**Conclusions:**

In conclusion, while overall VCA survival is high, outcomes have not improved over time, a trend likely driven by the introduction of higher-risk procedures such as uterus transplantation.

**Applicability of Research to Practice:**

This research has direct clinical applicability by establishing benchmarks for patient and graft survival, allowing programs to evaluate their outcomes against national data. This work also provides a framework to guide patient management and observe the long-term functional success of VCA transplantation.

**Funding for the study:**

N/A.

